# Simvastatin Coadministration Modulates the Electrostatically Driven Incorporation of Doxorubicin into Model Lipid and Cell Membranes

**DOI:** 10.1021/acsbiomaterials.2c00724

**Published:** 2022-09-29

**Authors:** Aleksandra Bartkowiak, Ewa Nazaruk, Ewa Gajda, Marlena Godlewska, Damian Gaweł, Elżbieta Jabłonowska, Renata Bilewicz

**Affiliations:** † Faculty of Chemistry, University of Warsaw, Pasteura 1, 02093 Warsaw, Poland; ‡ Department of Biochemistry and Molecular Biology, Centre of Postgraduate Medical Education, Marymoncka 99/103, 01-813 Warsaw, Poland; § Department of Cell Biology and Immunology, Centre of Postgraduate Medical Education, Marymoncka 99/103, 01-813 Warsaw, Poland

**Keywords:** simvastatin, doxorubicin, cubosomes, Langmuir monolayer, cancer cells, MTS

## Abstract

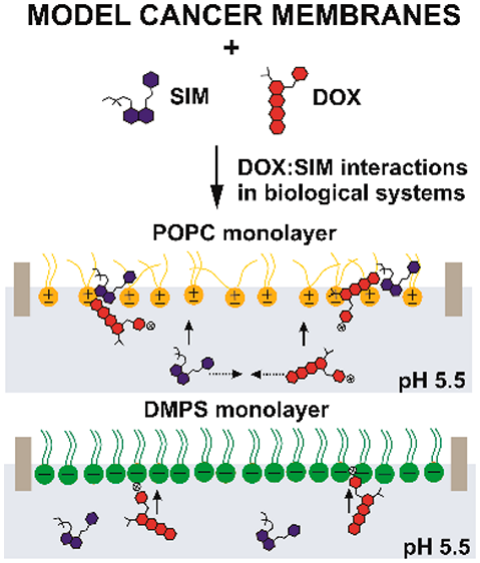

Understanding the interactions between drugs and lipid membranes is a prerequisite for finding the optimal way to deliver drugs into cells. Coadministration of statins and anticancer agents has been reported to have a positive effect on anticancer therapy. In this study, we elucidate the mechanism by which simvastatin (SIM) improves the efficiency of biological membrane penetration by the chemotherapeutic agent doxorubicin (DOX) in neutral and slightly acidic solutions. The incorporation of DOX, SIM, or a combination of them (DOX:SIM) into selected single-component lipid membranes, zwitterionic unsaturated 1-palmitoyl-2-oleoyl-*sn*-glycero-3-phosphocholine (POPC), neutral cholesterol, and negatively charged 1,2-dimyristoyl-*sn*-glycero-3-phospho-l-serine (DMPS) was assessed using the Langmuir method. The penetration of neutral lipid monolayers by the codelivery of SIM and DOX was clearly facilitated at pH 5.5, which resembles the pH conditions of the environment of cancer cells. This effect was ascribed to partial neutralization of the DOX positive charge as the result of intermolecular interactions between DOX and SIM. On the other hand, the penetration of the negatively charged DMPS monolayer was most efficient in the case of the positively charged DOX. The efficiency of the drug delivery to the cell membranes was evaluated under *in vitro* conditions using a panel of cancer-derived cell lines (A172, T98G, and HeLa). MTS and trypan blue exclusion assays were performed, followed by confocal microscopy and spheroid culture tests. Cells were exposed to either free drugs or drugs encapsulated in lipid carriers termed cubosomes. We demonstrated that the viability of cancer cells exposed to DOX was significantly impaired in the presence of SIM, and this phenomenon was greatly magnified when DOX and SIM were coencapsulated in cubosomes. Overall, our results confirmed the utility of the DOX:SIM combination delivery, which enhances the interactions between neutral components of cell membranes and positively charged chemotherapeutic agents.

## 1 Introduction

Simvastatin (SIM) is an antilipemic drug that promotes the inhibition of HMG-CoA reductase.^1^ Prior studies have shown that simvastatin monotherapy or combination therapy in a variety of cancers has shown beneficial results. SIM exerts its anticancer activity through a variety of mechanisms, particularly through the inhibition of angiogenesis, tumor cell proliferation and metastasis, and the induction of apoptosis in tumor cells.^2−4^ The effects of this drug depend on the cell line and the concentration and length of exposure to the drug.^5^ Simvastatin is particularly useful as an anticancer agent compared to other cytotoxic agents due to its low toxicity and minimal side effects.^2,3^

The use of nanocarriers loaded with anticancer drugs represents a promising strategy guaranteed to reduce toxicity to healthy tissues and increase the efficacy of chemotherapy in cancer treatment.^6^ Numerous *in vivo* and clinical studies have shown increased efficacy of SIM when placed in micro- and nanocarriers versus its administration in free form.^7−15^ Wu et al.^8^ reported that simvastatin-loaded star-shaped cholic acid–poly(d,l-lactide-*co*-glycolide) nanoformulations are more effective and exhibit increased sustained inhibition of breast adenoma growth than free SIM at the same dose. In another paper, Sedki et al.^9^ reported that the optimal PLGA-based hybrid nanocarrier significantly improves the antitumor activity of SIM against the prostate cancer cell line by interfering with the apoptosis mechanism and causing cell cycle arrest in the G2/M phase. In addition, Alhakamy et al.^10^ reported that SIM-loaded chitosan microparticles coated with Eudragit S100 formula exhibited improved colon targeting and enhanced cytotoxicity and proapoptotic activity against HCT-116 colon cancer cells. Long-circulation liposomes have been proposed as delivery systems for simvastatin in the treatment of C26 colon cancer. These liposomes have also been reported to have increased antitumor activity by increasing oxidative stress in the tumor environment. Liposomal treatment had an 85% greater inhibition of B16 melanoma cell growth compared with free simvastatin.^11−13^ The possibility of using immunoliposomes as SIM carriers has also been described.^14,15^ Recent studies showed the more potent *in vitro* antitumor activity of SIM-loaded cubosomes on MCF-7 cells compared to free SIM.^16^

Combinations of statins with other anticancer drugs could be very effective for cancer treatment and provide an alternative treatment option. Benefits of the combined use of SIM with cytotoxic doxorubicin (DOX) in anticancer therapy have been reported.^17−25^ DOX belongs to the class of anthracyclines and exhibits very potent activity against numerous types of cancer.^26^ However, DOX is also associated with severe and long-term side effects, including multiorgan toxicity.^27^ SIM is a promising option for potential association with DOX due to its lipophilic nature, which increases diffusion in cells.^28^ This increased diffusion is beneficial because it may increase the cytotoxic effect on cancer cells. No less important are statins, which have been shown to effectively mitigate DOX cardiotoxicity.^29,30^

The coadministration of statins and chemotherapeutic agents has recently been a subject of discussion. It was postulated that simvastatin could act synergistically with doxorubicin against cancer cells, probably through cell cycle regulation or the induction of apoptosis.^18,19,21−23^ In a recently published study,^24^ liposomes coencapsulating SIM and DOX were more detrimental to C26 mouse colon cancer cells cocultured with macrophages compared to their free forms. The cytotoxic effects of doxorubicin alone and in combination with simvastatin were found to depend on the ratio of drug concentrations in HeLa cell lines. The percent cell viability differed depending on whether DOX and SIM were coadministered and whether simvastatin was added to doxorubicin or vice versa after the incubation of the cells.^25^ The protocol of this combination therapy may therefore be important for achieving the optimal therapeutic results with these two drugs. Conversion of the lactone form of simvastatin to the corresponding hydroxy acid is strongly pH dependent. At physiological and alkaline pH, substantial proportions of simvastatin lactone are converted to the active hydroxy acid form. At slightly acidic pH, conversion occurs to a lower extent, resulting in a greater proportion of statin remaining in the more lipophilic lactone form.^31^ Thus, we performed the experiments at two pH’s, 5.5 and 9.0, to assess the efficacy of lipid layer penetration by the drugs individually and in combination.

Interactions between statins and anticancer agents could affect their penetration through biological membranes. Therefore, understanding these interactions may help to determine the optimal protocol for drug delivery. This motivated us to study the incorporation of SIM, DOX, or their mixture (DOX:SIM) into selected lipid membranes (Figure S1). Zwitterionic, unsaturated 1-palmitoyl-2-oleoyl-*sn*-glycero-3-phosphocholine (POPC), neutral cholesterol, and the negatively charged 1,2-dimyristoyl-*sn*-glycero-3-phospho-l-serine (DMPS) were chosen to form the model membranes since they constitute the external part of the cancer cell membrane.^32,33^ In order to understand the behavior in more complex biological systems, the interactions between drugs and single-component monolayers, which show only one selected property of a real biological membrane (POPC, fluidity; DMPS, negative charge), are very useful. The Langmuir monolayer characteristics (e.g., increased area per molecule, changed collapse pressure, or modified compressibility factor) allow one to pinpoint the factor modulating the drug incorporation, e.g., the charge of the drug or its hydrophobicity, the charge of the lipid building the monolayer, or the interaction between drugs in the combined drug delivery. The facility of DOX and SIM incorporation into the selected single-component model membranes was found to depend both on the charge of the lipid monolayer membranes and on the pH-dependent charges of the drugs.

The MTS and trypan blue exclusion assays were used to assess the differences in the toxicity of the free drug (DOX, SIM, DOX:SIM) and also of the drugs administered in the form of cubic phase nanoparticles (MO/DOX, MO/DOX:SIM) toward the malignant glioma cell lines A172 and T98G as well as the HeLa cell line.^34−36^

## 2 Materials and Methods

### 2.1 Materials

Monoolein (MO; 1-oleoyl-rac-glycerol; purity ≥99%) and Pluronic F108 (PF108) used for mesophase synthesis were purchased from Sigma-Aldrich. The lipids used in the experiments, 1-palmitoyl-2-oleoyl-*sn*-glycero-3-phosphocholine (POPC), 1,2-dimyristoyl-*sn*-glycero-3-phospho-l-serine (sodium salt) (DMPS), and cholesterol (Chol), were of high purity (≥99%) and purchased from Avanti Polar Lipids. Stock solutions were prepared by dissolving either POPC and cholesterol in chloroform or DMPS in a 4:1 v/v chloroform/methanol mixture. HPLC grade organic solvents were purchased from Sigma-Aldrich. The subphase used in the Langmuir experiments was a MES buffer solution (0.01 M; pH 5.5; Sigma-Aldrich) and TRIS buffer solution (0.01 M; pH 9.0; Sigma-Aldrich) or buffer solutions with doxorubicin hydrochloride (DOX) (AK Scientific) and/or simvastatin (lactone) (SIM) (Sigma-Aldrich) at a concentration of 10^–6^ M for both DOX and SIM. The buffer solutions were prepared using Milli-Q water with a resistivity of 18.2 MΩ·cm.

### 2.2 Preparation of Drug Loaded Lipid Liquid Crystalline Nanoparticles

Cubosomes were prepared according to a slightly modified protocol that has been previously described by our group.^35−37^ In order to produce cubosomes, simvastatin and doxorubicin were dissolved in DMSO and added to molten monoolein (at 40 °C). The mole ratio of MO/DOX:SIM in the cubosome dispersion was 88/10:2 mol %. The homogeneous lipid mixture was hydrated by a solution of stabilizer, Pluronic F108 (5 mg/mL). The sample was homogenized using SONICS Vibracell VCX 130 (Sonics & Materials Inc.) at 40% for 20 min (2 s sonic pulses interrupted by 3 s breaks).

The phase identity of the mesophases was determined by small-angle X-ray scattering (SAXS) (Bruker Nanostar system working with Cu Kα radiation equipped with a Vantec 2000 area detector).^35−37^ All of the measurements were performed in 1.5 mm capillaries. The samples were measured at 25 °C. Prior to measurement, dispersions were left overnight to equilibrate at room temperature. Diffraction rings were observed upon exposure to X-rays, which were further used to distinguish the phase. The 2D pattern was integrated into a 1D scattering function *I*(*q*) (where *q* (nm^–1^) is the length of the scattering vector). The scattering vector (*q*) values of the peaks were correlated with Miller indices for known mesophases to identify the phase type. The cubic phase of the *Pn*3*m* symmetry shows the *q* values correspond to the scattering peaks in the ratio of √2:√3:√4:√6:√8:√9.

### 2.3 Langmuir Technique

Experiments were carried out using a computer controlled KSV Nima Langmuir balance (Biolin Scientific, Uppsala) equipped with a Langmuir trough (total area = 587 cm^2^) and two hydrophilic barriers that allowed symmetric compression of the liquid surface. A Wilhelmy plate (filter paper) was used as a surface pressure sensor. After cleaning the trough with chloroform and methanol and rinsing with plenty of water, the trough used for the monolayer preparation was filled with either buffer alone or buffer containing different concentrations of DOX, SIM, and their mixture (DOX:SIM). After spreading the lipid solution on the subphase, the solvent was allowed to evaporate for 10 min. The spreading film was compressed symmetrically from both sides at a constant rate of 10 mm/min (7.5 cm^2^/min), and the surface pressure (π) vs area per molecule (*A*) isotherm was simultaneously recorded. All the experiments were performed at 21 ± 1 °C.

On the basis of the surface pressure–area per molecule isotherms (π–*A*), the following parameters were determined: lift-off area (*A*_lift-off_), limiting area (*A*_0_), mean area at surface pressures, π in the range of 10–45 mN/m (*A*_π [mN/m]_), and compressibility modulus (*C*_s_^–1^). The parameter *A*_lift-off_ defines the threshold at which the transition from the gas phase to the expanded liquid occurs. In other words, this parameter refers to the level at which the isotherm begins to rise. The limiting area *A*_0_ is obtained by extrapolating the linear part of the isotherm to zero surface pressure.

Changes in the phase and, thus, in the orientation of the lipid molecules and the presence of phase transitions can be followed by the changes in the values of the compressibility modulus (*C*_s_^–1^), which is defined as^38^
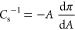
1

This parameter gives information on the phase of the monolayer at a given surface pressure, while any minimum in a *C*_s_^–1^ vs surface pressure plot corresponds to a phase transition occurring in the monolayer. The states of the monolayers are classified on the basis of the maximal values of *C*_s_^–1^ in the plots of *C*_s_^–1^ versus π in the following way: max*C*_s_^–1^ values are in the range of 12.5–50 mN/m for the liquid expanded (LE) films, max*C*_s_^–1^ = 50–250 mN/m for the liquid condensed (LC) film, and max*C*_s_^–1^ values above 250 mN/m the monolayer are classified as a solid (S) film.^38^

In order to quantify the interaction of DOX, SIM, and DOX:SIM with the monolayer, the increase in surface area (Δ*A*) for each system was determined according to eqs 2–4:
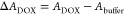
2
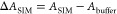
3
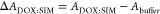
4where *A*_buffer_, *A*_DOX_, *A*_SIM_, and *A*_DOX:SIM_ are the areas of each molecule at a given surface pressure (π) of the monolayer formed on a pure buffer and buffer with simvastatin, doxorubicin, or both drugs in the subphase, respectively. The obtained data (Tables 1–3) can be used to determine whether or not there is synergy in the interaction of the two drugs with the tested monolayers.

**Table 1:** Increase in the Molecular Area Read at π = 20 mN/m for the POPC Monolayer Formed on MES Buffer (pH 5.5) and TRIS Buffer (pH 9.0) Containing DOX (10^–6^ M), SIM (10^–6^ M), and DOX:SIM Molecules in a 1:1 Molar Ratio

	molecular area read at π = 20 mN/m	
subphase [Å^2^/molecule]	MES pH 5.5	TRIS pH 9.0
Δ*A*_DOX (1×10^–6^)_	1.7 ± 1.0	6.5 ± 1.1
Δ*A*_SIM (1×10^–6^)_	41.8 ± 1.5	9.8 ± 1.6
Δ*A*_DOX:SIM (1:1)_	48.3 ± 1.5	14.9 ± 1.8

### 2.4 Cell Lines and Culture Conditions

Glioblastoma-derived cell lines A172 and T98G and cervical cancer derived HeLa cells were obtained from the American Type Culture Collection (ATCC; Manassas, VA, USA). All cell lines were grown in high glucose Dulbecco’s modified Eagle’s medium (DMEM; Corning, New York, NY, USA) supplemented with 10% fetal bovine serum (FBS; HyClone, Cytiva, Marlborough, MA, USA) and 1% antibiotic–antimycotic solution (Sigma-Aldrich, Steinheim, Germany). Cultures were maintained at 37 °C in a humidified atmosphere of 5% CO_2_. The ratio of DOX:SIM (using free compounds or both drugs introduced to cubosomes) in the case of cell culture experiments was 1:16.

### 2.5 Cell Viability (Trypan Blue Exclusion Assay)

The trypan blue exclusion staining technique was used to differentiate viable from nonviable cells as previously described^39^ with a few minor modifications

Briefly, 1 mL of cell suspension (1 × 10^5^ cells) in complete medium was seeded into each well of a 12-well plate. The next day, the culture medium was supplemented with free SIM (5 × 10^–2^ M) and/or free DOX (3 × 10^–6^ M), empty MO-based cubosomes (3.57 μL/mL), DOX-loaded MO-based cubosomes (3.57 μL/mL; DOX concentration of 3 × 10^–6^ M), or DOX- and SIM-loaded MO-based cubosomes (3.57 μL/mL; DOX and SIM concentrations of 3 × 10^–6^ and 5 × 10^–2^ M, respectively), and the cells were incubated for an additional 24 and/or 48 h. Untreated cells were used as controls. The attached and detached cells were then harvested, pelleted, and resuspended in Dulbecco’s phosphate buffered saline (D-PBS; HyClone, Cytiva, Marlborough, MA, USA) and stained with trypan blue (NanoEnTek Inc., Seoul, Korea). The percentage of viable cells collected in the total cell population was determined using an EVE automated cell counter (NanoEnTek Inc., Seoul, Korea).

### 2.6 Cell Viability (MTS Assay)

The viability of the cells was estimated on the basis of the activity of mitochondrial dehydrogenases using a tetrazolium compound assay (CellTiter 96 AQueous One Solution Cell Proliferation MTS Assay; Promega, Madison, WI, USA) as previously described^36^ with some minor modifications. Briefly, 3 × 10^3^ cells were suspended in 100 μL of complete growth medium and seeded into the wells of a 96-well plate. The next day, media were supplemented with empty, DOX-loaded, or DOX- and SIM-loaded cubosomes, as described above. Untreated cells served as controls. After 24 or 48 h, 20 μL of the MTS reagent was added to each well and the incubation was continued for another 3 h. Absorbance was recorded at 490 and 650 nm using the Synergy 2 reader (BioTek Instruments, Winooski, VT, USA). The results were expressed as the percentage of proliferating cells compared to the untreated controls.

### 2.7 Fluorescence Imaging

Confocal imaging was used to determine DOX accumulation and cell viability as previously described^40^ with a few minor modifications. Briefly, T98G (1 × 10^5^) and HeLa (1 × 10^5^) cells were seeded on uncoated cover glasses in a 6-well plate and in 2 mL of complete medium. The next day, the growth medium was supplemented with empty, DOX-loaded, or DOX- and SIM-loaded cubosomes as described above, and the incubation was continued for an additional 24 h (HeLa) or 48 h (T98G). Untreated cells were used as controls. Harvested cover glasses were fixed for 10 min with 4% paraformaldehyde (Sigma-Aldrich, Steinheim, Germany) in PBS (pH 7.4) and permeabilized for 10 min with 0.25% Triton X-100 (Sigma-Aldrich, Steinheim, Germany) in Milli-Q water. Next, the cells were blocked with 2% bovine albumin in TRIS-buffered saline (TBS; pH 8.0) containing 0.1% Tween 20 (TBS-T) for 1 h followed by a 30 min incubation with FITC-conjugated phalloidin (2 μg/mL; Sigma-Aldrich, Steinheim, Germany) in PBS (pH 7.4) and a 2 min incubation with 4′,6-diamidino-2-phenylindole (DAPI; 0.4 μg/mL; Sigma-Aldrich, Steinheim, Germany) in Milli-Q water. After each step, the cells were washed with PBS (pH 7.4). The cells were mounted with Fluorescence Mounting Medium (Dako) and examined using the Zeiss LSM800 confocal unit equipped with a plan-apochromatic 63×/1.4 oil DIC M27 lens (Carl Zeiss, Oberkochen, Germany).

### 2.8 Spheroid Formation Assay

The growth of the spheroids in response to the nanoparticles was assessed as previously described.^41^ HeLa cells (5 × 10^3^) were seeded on an ultralow attachment 96-well round-bottom plate (Corning, New York, NY, USA) in 100 μL of complete medium. The next day, the culture medium was supplemented with empty, DOX-loaded, or DOX- and SIM-loaded cubosomes as described above. Untreated spheroids were used as a control. After 12 days of incubation, the images were captured using the Observed D1 microscope (10 lens; Carl Zeiss, Oberkochen, Germany) equipped with AxioVision LE software (Carl Zeiss, Oberkochen, Germany). The areas of the spheroids were quantitated using the ImageJ software (NIH, Bethesda, MD, USA).

### 2.9 Statistical Analysis

Biological data were analyzed using GraphPad Prism 6.0 for Windows (GraphPad, Inc., San Diego, CA, USA) and expressed as the mean ± standard deviation (SD). For statistical analyses, the normality of the data was confirmed using the Shapiro–Wilk test, followed by the one-way ANOVA and the Bonferroni post hoc comparative test. Results were considered statistically significant at *p*-values below 0.05.

## 3 Results and Discussion

### 3.1 Langmuir Monolayer Studies of the Effect of the DOX, SIM, and Their Mixture DOX:SIM on the Model Cancer Cell Membrane Formed at the Air–Water Interface

Langmuir isotherms provide information regarding the interactions between drugs and lipids at the air–water interface that are important for understanding the drug’s ability to penetrate the lipid layer and affect the monolayer properties. In this regard, monolayers composed of zwitterionic (POPC), negatively charged (DMPS), and uncharged (cholesterol) lipids were used as simple models to study the effect of simvastatin, doxorubicin, and their mixture DOX:SIM at the membrane surface level. The changes in the surface pressure area per molecule (π–*A*) isotherm shapes and characteristic parameters of the monolayers due to the interactions with the drugs were followed.

**Figure 1:**
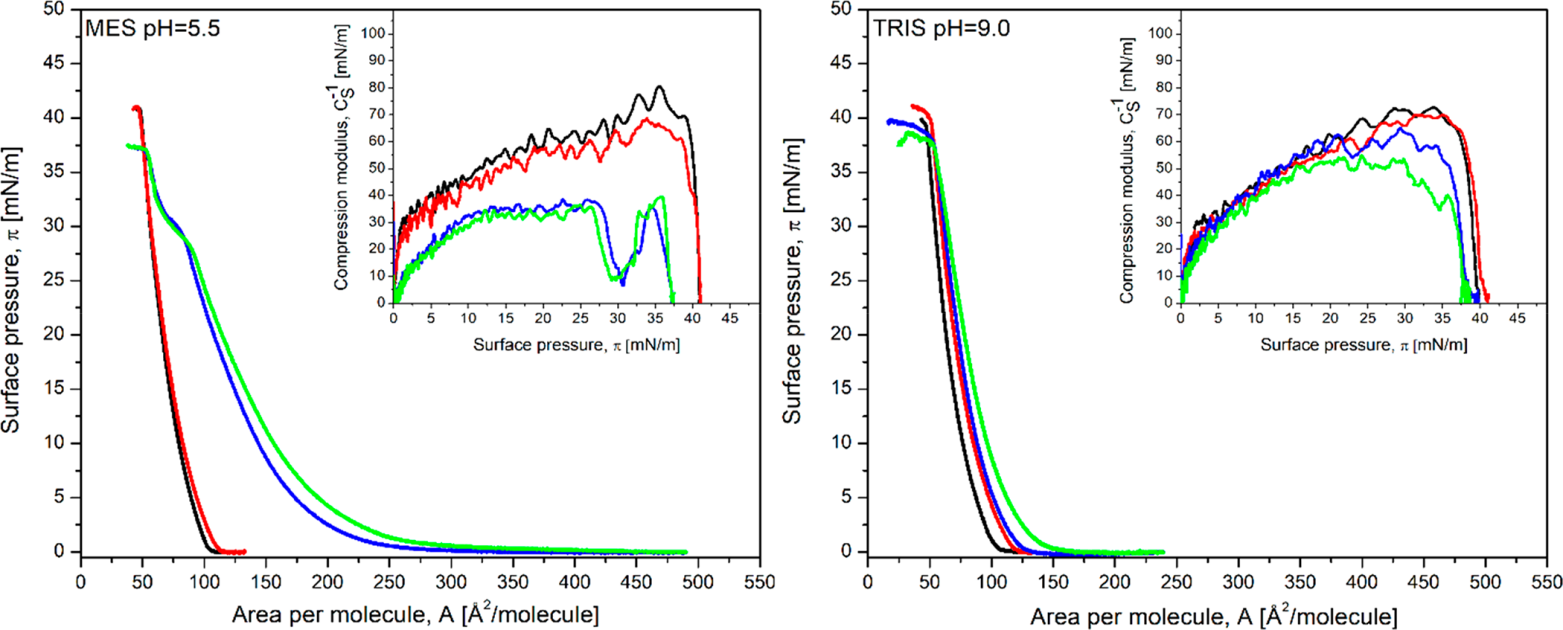
Surface pressure (π)–area per molecule (*A*) isotherms of the POPC monolayer formed on buffer solution (black line) and buffer containing doxorubicin (DOX) at a concentration of 10^–6^ M (red line), simvastatin (SIM) at a concentration of 10^–6^ M (blue line), and their mixture (DOX:SIM) in a molar ratio of 1:1 (green line). Insets: compression modulus versus surface pressure plot of the POPC monolayer.

**Figure 2:**
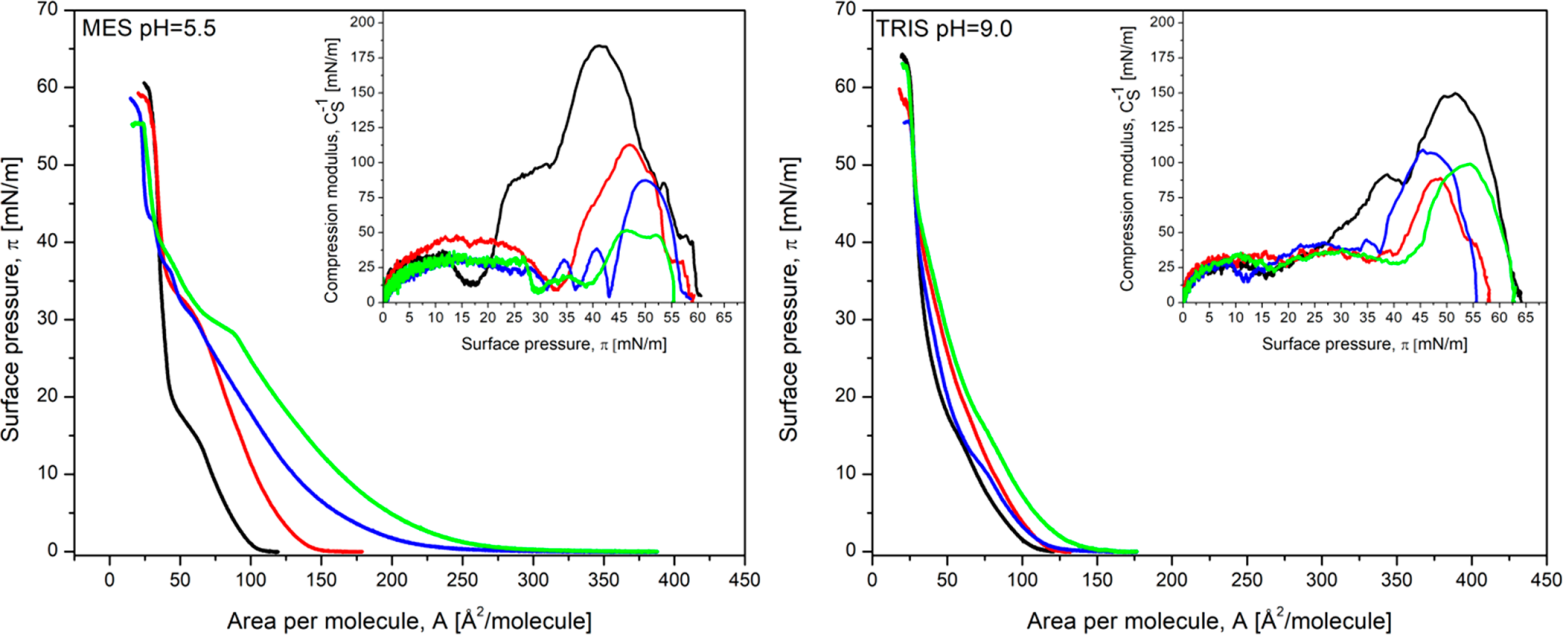
Surface pressure (π)–area per molecule (A) isotherms of the DMPS monolayer formed on buffer solution (black line) and buffer containing DOX at a concentration of 10^–6^ M (red line), SIM at a concentration of 10^–6^ M (blue line), and their mixture (DOX:SIM) in the molar ratio of 1:1 (blue line). Insets: compression modulus versus surface pressure plot of the DMPS monolayer.

**Figure 3:**
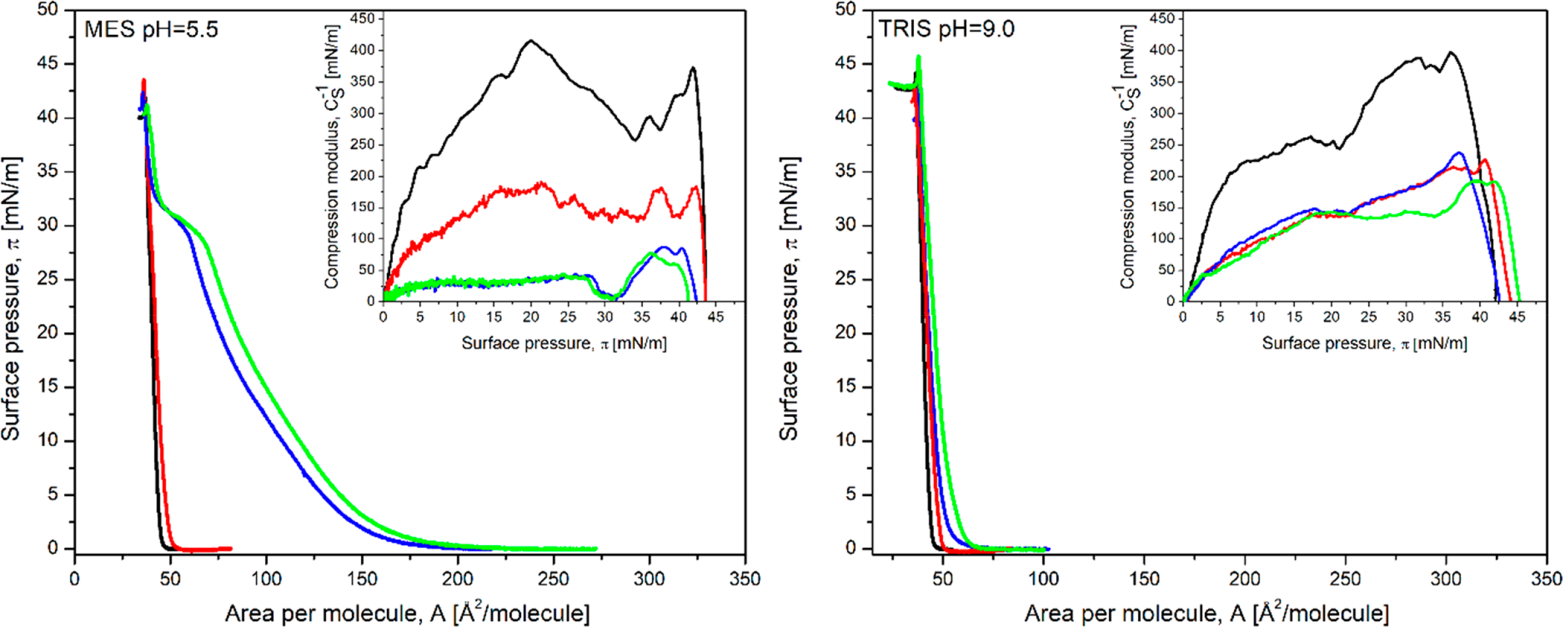
Surface pressure (π)–area per molecule (A) isotherms of the Chol monolayer formed on buffer solution (black line) and buffer containing DOX at a concentration of 10^–6^ M (red line), SIM at a concentration of 10^–6^ M (blue line), and their mixture (DOX:SIM) in the molar ratio of 1:1 (green line). Insets: compression modulus versus surface pressure plot of the Chol monolayer.

The π–*A* isotherms of POPC, DMPS, and cholesterol (Chol) monolayers were recorded for the pure MES buffer (pH 5.5) and TRIS buffer (pH 9.0) subphases and for the subphases containing doxorubicin (concentration 10^–6^ M), simvastatin (concentration 10^–6^ M), and their mixture (DOX:SIM) in a 1:1 molar ratio (10^–6^ M:10^–6^ M) (Figures 1–3). The parameters of the π–*A* isotherms for POPC, DMPS, and Chol Langmuir monolayers exposed to solutions of DOX, SIM, and their mixture DOX:SIM are presented in Tables S1, S3, and S5. The characteristics of π–*A* isotherms recorded for monolayers composed of pure membrane lipids at the air–water interface are very well-known, and our results are in agreement with previously published data (for POPC,^42,43^ DMPS,^44^ and cholesterol^43,45^). Moreover, the presence of individual molecules of DOX and SIM and their mixture (DOX:SIM) in the subphase leads to a shift of isotherms toward higher surface area per molecule values compared to isotherms recorded for POPC, DMPS, and Chol formed on pure buffer subphases, indicating the presence of both drugs at the air–water interface. The values of the area at which the isotherm lift-off point appears are included in Tables S1, S3, and S5.

POPC is a zwitterionic lipid, and the film prepared on pure MES (pH 5.5) or TRIS (pH 9.0) buffer is in a liquid-condensed phase. On the basis of the π–*A* isotherms (Figure 1) and the maximum values of compressibility modulus (max*C*_s_^–1^, eq 1) (Table S1), a very strong interaction can be observed between the phospholipid (POPC) and SIM present in the subphase at pH 5.5.

Moreover, there is a characteristic plateau on the isotherms at a surface pressure of 30 mN/m, at which reorganization of the layer occurs due to the incomplete expulsion of the drug molecules from the POPC monolayer (Figure 1). In addition, the presence of SIM molecules in the subphase results in a reduction in max*C*_s_^–1^ values from about 77 to 37 mN/m and a transition of the monolayer to the liquid-expanded phase using the criteria of Davies and Rideal.^38^ This agrees well with our previous research^46^ showing that SIM has a strong fluidizing effect on the DMPC monolayer. Simvastatin molecules have a negative charge at pH 9.0 and are uncharged at pH 5.5. In the case of measurements conducted at pH 9.0, the interaction of SIM molecules with the POPC membrane is, therefore, weak (Figure 1), and the POPC membrane remains in the liquid-condensed phase.

Doxorubicin is a weak acid with a p*K*_a_ value of 8.2. Under acidic conditions, the protonated amino group of doxorubicin is positively charged. The POPC monolayer formed on MES buffer (pH 5.5) interacts very weakly with the positively charged DOX ions at this pH present in the subphase (only small shifts toward higher values of surface area per molecule were observed (Figure 1)). In contrast, at pH 9.0, the hydrophobic interactions between the neutral DOX molecules and the POPC monolayer lead to a larger shift of the isotherm toward higher surface areas per molecule, which reflects a facilitated incorporation of the drug into the monolayer compared to the behavior observed at pH 5.5.

The data collected in Table 1 suggest that, at a pH of 5.5, the largest increase in surface area values is observed when both drugs are present in the subphase. These effects are even more pronounced at lower surface pressures (Figures S2 and S6). It indicates that strongly lipophilic and neutral SIM facilitates the incorporation of positively charged DOX into the neutral membrane.

At pH 9.0, the simultaneous incorporation of both drugs into the layer is less favorable. The statin in its negatively charged form clearly does not show the unique affinity for the POPC monolayer exhibited under acidified solution conditions (Table 1).

The second lipid membrane used in our investigations of interactions with the drugs was the DMPS monolayer. The phospholipid has a negative charge due to the presence of a serine residue in the polar headgroup (Figure S1). DMPS monolayers recorded on MES and TRIS buffers show relatively large values of compressibility modulus corresponding to the liquid-condensed state of the monolayer (Figure 2 and Table S3).

At acidic pH, the presence of DOX in the subphase induces changes in the shape and properties of the isotherm (Table S3 and Figure 2). These data are consistent with numerous literature reports that doxorubicin interacts strongly with phosphatidylserine.^44,47,48^ This reflects the role of charges in the interactions of the drug with the lipid membrane. Strong electrostatic attractive interactions of positively charged DOX with the negatively charged membrane determine the ease of incorporation of this drug.

As illustrated in Figure 2, the presence of SIM molecules in the subphase at a pH of 5.5 strongly affects the shape of the π–*A* isotherm recorded for the DMPS monolayer. The phase transition occurs at higher surface pressures (π) and the layer becomes less condensed as shown by the reduction in max*C*_s_^–1^ values (from approximately 186 to 75 mN/m at pH 5.5) At higher surface pressures (>40 mN/m), the drug is squeezed out of the layer.

Because DOX itself interacts very strongly with DMPS, the presence of both drugs in the subphase does not further increase their penetration into the layer. Weaker interactions with SIM do not play any role in strengthening the simultaneous membrane penetration by both drugs in solutions of pH lower than the p*K*_a_ of DOX. At high pH, the interactions between both the neutral DOX and negatively charged SIM with the DMPS monolayer are much weaker (Table 2). When the drugs are delivered together, their incorporation is slightly more efficient than when they are individually present in the subphase. This allows us to suggest that the efficiency of membrane penetration depends on the interplay between the penetration efficiency of the single components and the effects of the complex formation in the solution.

**Table 2:** Increase in the Molecular Area Read at π = 20 mN/m for the DMPS Monolayer Formed on MES Buffer (pH 5.5) and TRIS Buffer (pH 9.0) Containing DOX (10^–6^ M), SIM (10^–6^ M), and DOX:SIM Molecules in a 1:1 Molar Ratio

	molecular area read at π = 20 mN/m	
subphase [Å^2^/molecule]	MES pH 5.5	TRIS pH 9.0
Δ*A*_DOX (1×10^–6^)_	38.0 ± 0.9	13.3 ± 1.3
Δ*A*_SIM (1×10^–6^)_	48.4 ± 2.1	4.2 ± 1.2
Δ*A*_DOX:SIM (1:1)_	73.5 ± 1.7	18.0 ± 1.5

The third model membrane used to study the interactions with the drugs separately and together in the subphase is a well-organized cholesterol monolayer characterized by high max*C*_s_^–1^ (400 mN/m) values (Figure 3 and Table S5). A significant change in the isotherm shape is observed when SIM is present in the subphase while DOX penetration is almost negligible and can be detected only by the lower compressibility modulus (Table 2 and Figure S5).

The shape of the isotherm indicates that, at a pH of 5.5, the efficient incorporation of SIM molecules causes strong fluidization of the Chol monolayer and its transition from a solid to the liquid-condensed phase (inset in Figure 3, Table S5). Sharp minima appear in the max*C*_s_^–1^ vs surface pressure plots at a surface pressure of 30 mN/m (inset in Figure 3). A detailed analysis of the changes in the properties of the π–*A* isotherms recorded for the Chol monolayer due to SIM incorporation are described in our previous work.^46^ In turn, at a pH of 9.0, negatively charged SIM interacts very weakly with the Chol monolayer and almost no changes of the Chol isotherm are seen.

In the presence of DOX:SIM (1:1), sharp minima were observed at a surface pressure of 30 mN/m in the *C*_s_^–1^ vs surface pressure plots (inset in Figure 3). In general, such minima indicate orientation changes or phase transitions The simultaneous presence of both drugs in the subphase results in a decrease in the compression modulus to ∼30 mN/m. The results collected in Table 3 show that the simultaneous presence of both drugs in the solution leads to their increased incorporation into the cholesterol monolayer similarly to the behavior observed in the case of the POPC monolayer. At a pH of 9.0, both drugs are not as efficiently incorporated into the layer, and the max*C*_S_^–1^ values indicate that the monolayers in the presence of DOX and SIM are in the liquid-condensed phase. This is not unexpected due the negative charge of SIM and its weak interaction with the neutral cholesterol monolayer. Still, some fluidization of the cholesterol layer can be recognized.

**Table 3:** Increase in the Molecular Area Read at π = 20 mN/m for the Chol Monolayer Formed on MES Buffer (pH 5.5) and TRIS Buffer (pH 9.0) Containing DOX (10^–6^ M), SIM (10^–6^ M), and DOX:SIM Molecules in a 1:1 Molar Ratio

	molecular area read at π = 20 mN/m	
subphase [Å^2^/molecule]	MES pH 5.5	TRIS pH 9.0
Δ*A*_DOX (1×10^–6^)_	1.8 ± 1.5	6.3 ± 1.7
Δ*A*_SIM (1×10^–6^)_	35.7 ± 2.5	2.8 ± 0.8
Δ*A*_DOX:SIM (1:1)_	44.6 ± 2.0	3.8 ± 2.0

As shown above at pH 5.5, the DOX molecules alone show very weak interactions with the Chol monolayer as opposed to SIM; therefore, they are effectively introduced into the membrane in the presence of SIM, which again points to the electrostatically modulated DOX penetration. Interactions with SIM neutralize the charge effect of DOX and facilitate its incorporation into the neutral cholesterol layer. On the other hand, at pH 9.0, SIM incorporation is not favorable; therefore, the insertion of both drugs together is also not effective (Table 3).

In a different approach, the drugs were delivered to the subphase already covered by preformed membrane monolayers and the changes of surface pressure were observed over time (Figures S2–S4). The monolayers of POPC, DMPS, and cholesterol were first formed by compression to the surface pressure of 20 mN/m, and then, solutions of DOX, SIM, or DOX:SIM (1:1 molar ratio) were injected into the subphase under the monolayer. The increase in the surface pressure measured after 4 h (Tables S7–S9) confirms the facilitated drugs were incorporated after the injection of DOX:SIM in a 1:1 molar ratio to the subphase at pH 5.5. This indeed strengthens our hypothesis on the utility of combined drug delivery in the case of solutions with a pH below the p*K*_a_ of DOX. The strong affinity of SIM for all of the studied layers at acidic pH facilitates the incorporation of the positively charged DOX under conditions when its individual penetration into neutral membranes is not as efficient.

These observations led us to perform biological tests in order to establish the best way to deliver both drugs into the cancer cells. The effect of treatment of the designated model cell lines with single drugs and their combination was assessed. The analysis was further expanded through the usage of the lipid drug carriers, cubosomes coloaded with DOX and SIM. This allows one to deliver higher concentrations of drugs to the affected cells.

### 3.2 Biological Studies in the Presence of Drugs Delivered Individually and in Combination

**Figure 4:**
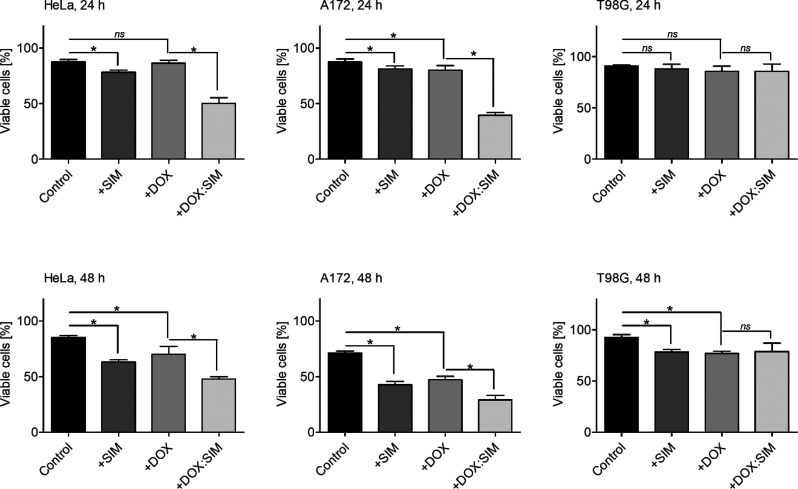
Viability of HeLa, A172, and T98G cells treated for 24 and 48 h with free SIM (+SIM), free DOX (+DOX), and the DOX:SIM cocktail (+DOX:SIM) determined by the trypan blue exclusion assay. Cotreatment with DOX and SIM results in the enhanced reduction of HeLa and A172 cells’ viability. Nontreated cells served as controls. Data are presented as mean ± standard deviation (SD). *p < 0.05. One-way ANOVA followed by the Bonferroni post hoc comparative test were performed.

**Figure 5:**
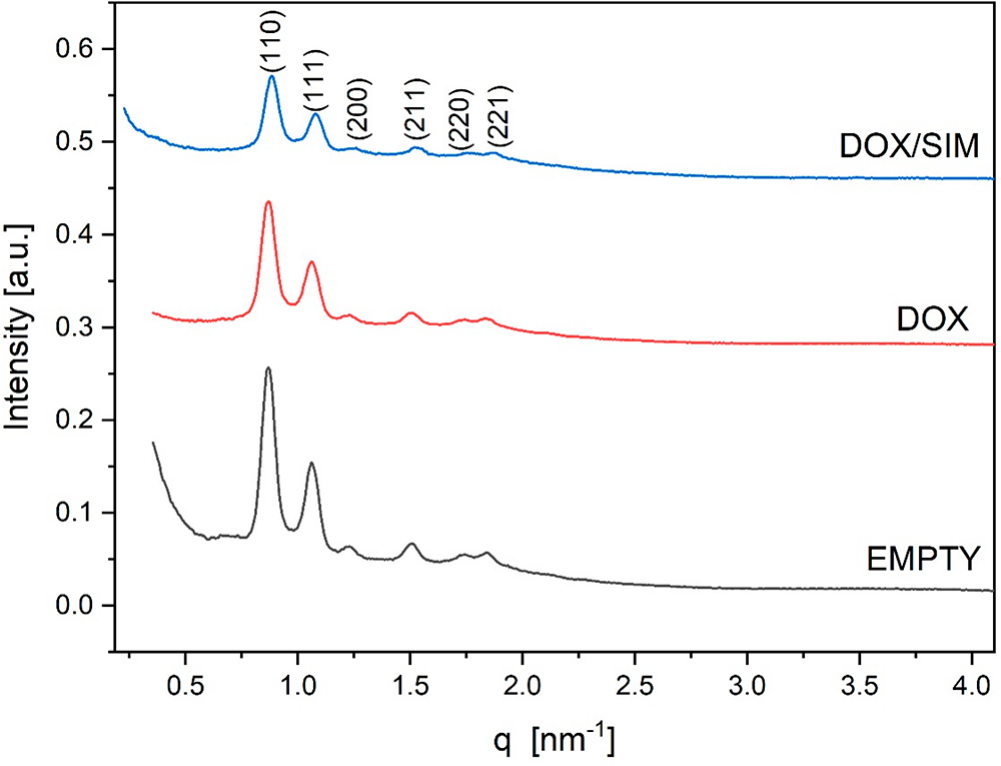
SAXS diffraction patterns obtained for DOX:SIM-loaded cubosomes. Miller indices [*hkl*] are shown for Bragg reflections for the cubic phase. The Bragg peaks correspond to the [110], [111], [200], [211], [220], and [221] reflections of a double diamond *Pn*3̅*m* cubic phase.

To evaluate the effect of SIM on the drugs’ accessibility of tumor cells, both glioma-derived (A172, drug-sensitive; T98G, drug-resistant) and HeLa cell lines were used. First, the effect of the free drugs on cell viability was evaluated. Cultured lines were exposed to both drugs separately and in combination, as described in the Materials and Methods. As shown in Figure 5, free SIM and free DOX did not affect the viability of any of the tested cell lines at the applied concentrations (5 × 10^–5^ and 3 × 10^–6^ M, respectively). Interestingly, it was observed that the DOX:SIM cocktail significantly reduced the survival rates of A172 and HeLa cells by nearly 2-fold. This strong antitumor effect of the drug in the presence of SIM is apparently a result of the increased effectiveness of drug shuttling due to the presence of SIM in the medium. As expected, the strong affinity of SIM for the cell membrane balances the positive charge of DOX, resulting in enhanced penetration of the cellular lipid barrier (as discussed above). The viability of T98G cells exposed to DOX:SIM was found to be unaffected (Figure 4). This finding is consistent with our previous observations^41^ and is likely the consequence of the strong, naturally acquired drug resistance exhibited by this cell line.

### 3.3 Biological Studies in the Presence of Drugs Encapsulated in Cubosomes

The potential effect of DOX and SIM on cancer-derived cells was further explored using MO-based cubosomes, which facilitate the encapsulation of various agents and act as carriers.^34−37^

To elucidate the structure of the formulations, we conducted SAXS measurements.^35−37^Figure 5 shows the typical SAXS patterns for the obtained formulations. The SAXS diffraction patterns for DOX:SIM-loaded cubosomes exhibit a sequence of diffraction peaks with relative positions at a ratio of √2:√3:√4:√6:√8, which can be attributed to the double diamond (*Pn*3̅*m*) cubic symmetry with a lattice parameter (*a*) of 10.3 nm. The SAXS profile of the nondoped monoolein cubosomes was displayed as a control to show that no obvious change after coloading SIM and DOX together into the cubosomes was observed.

**Figure 6:**
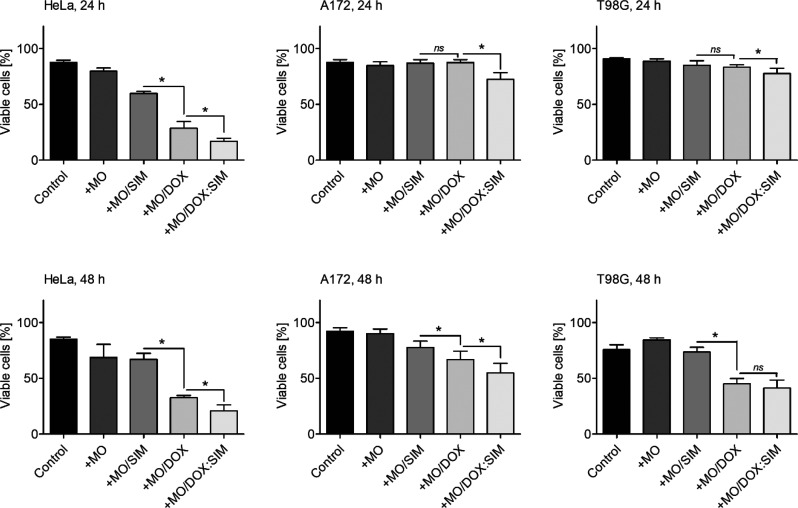
Viability of HeLa, A172, and T98G cells treated for 24 h (upper panel) and 48 h (lower panel) with empty MO-based cubosomes (+MO), DOX-loaded MO-based cubosomes (+MO/DOX), and DOX:SIM-loaded MO-based cubosomes (+MO/DOX:SIM) determined by the trypan blue exclusion assay. Treatment with DOX:SIM MO-based cubosomes results in a strong reduction in cell viability. Nontreated cells served as controls. Data are presented as mean ± SD; *p < 0.05. One-way ANOVA followed by the Bonferroni post hoc comparative test were performed.

As previously shown, MO-based cubosomes have relatively low toxicity and high cargo loading capacity and stability and are considered a functional drug-shuttle system.^35,37^ Cubosomes likely fuse with the plasma bilayer membrane and release cargo in contact with the cellular membrane.^36,49^ It was confirmed that cargo-free phases do not affect survival rates. DOX-loaded cubosomes reduced the cell viability of all the tested cells, including the drug-resistant T98G cells, after 24 and 48 h of treatment (Figures 6 and S5; trypan blue and MTS-based assay data, respectively). The toxicity of DOX varied and was most noticeable for HeLa and A172 cells. Most importantly, cells exposed to the newly formed DOX:SIM-loaded phases presented an even greater sensitivity to the chemotherapeutic, as SIM facilitated the fusion of DOX-loaded cubosomes with cell membranes. This resulted in more efficient DOX delivery. The antitumor effect of the DOX:SIM-loaded cubosomes was observed for all tested cells at both time points.

**Figure 7:**
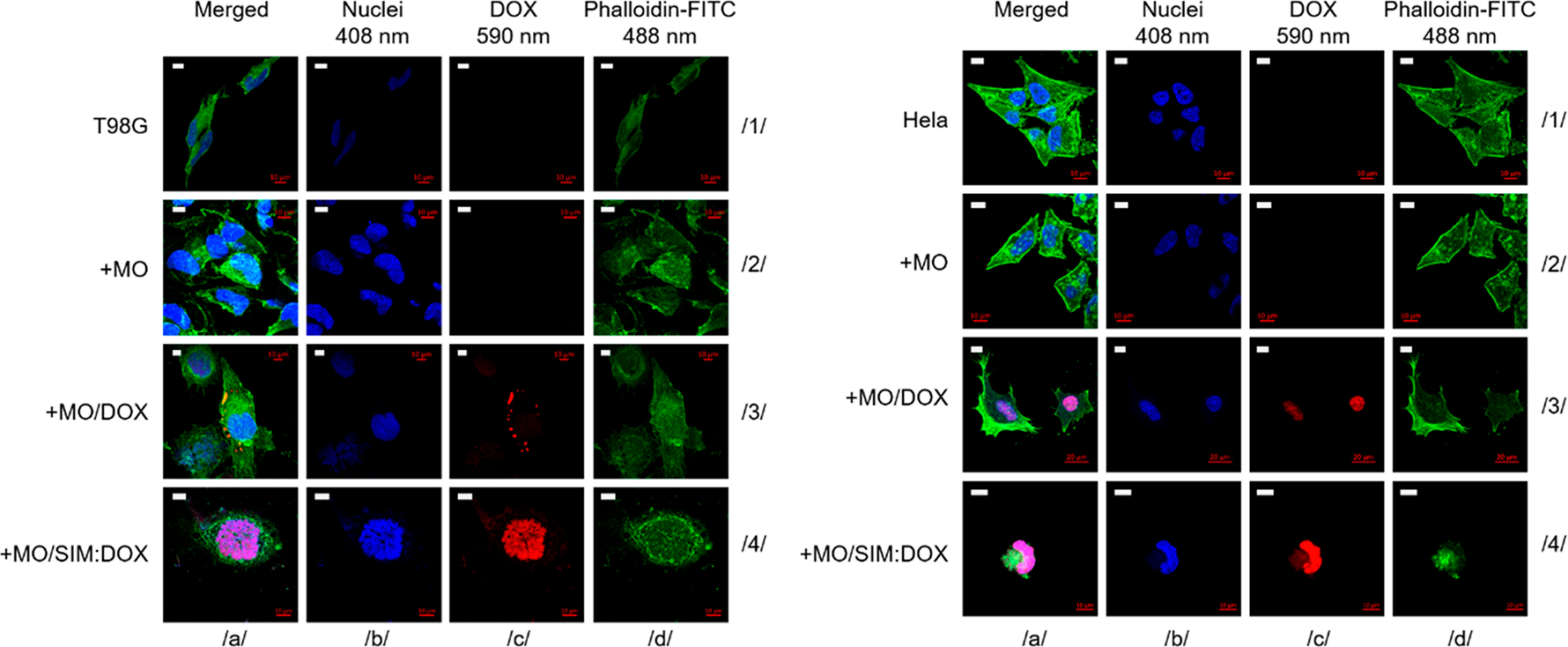
Confocal imaging of T98G (left panel) and HeLa (right panel) cells treated for 24 h (HeLa) or 48 h (T98G) with empty MO-based cubosomes (+MO; row 2), DOX-loaded MO cubosomes (+MO/DOX; row 3) and DOX:SIM-loaded MO cubosomes (+MO/DOX:SIM; row 4). Nontreated cells served as controls (T98G and HeLa; row 1). Blue fluorescence signal, DAPI staining of the nucleus; red fluorescence signal, DOX accumulation; green fluorescence signal, cytoskeleton staining using phalloidin conjugated with FITC. Scale bar: 10 μm.

Additionally, confocal imaging-based analysis was performed to confirm the observed combined effect of SIM and DOX on cultured cells. Figure 7 summarizes the performed analysis. As expected, cubosomes loaded together with DOX and SIM most effectively deliver chemo drugs into cells (measured as the intranuclear intensity of the red signal) and kill them (Figure 7, row 4). In contrast, only minor alternations in the shape and organization of the cells treated with empty phases or carriers loaded with DOX were noticed (Figure 7, rows 2 and 3, respectively).

**Figure 8:**
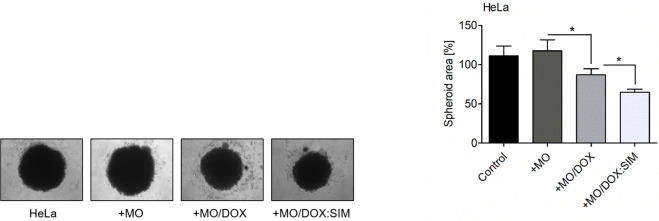
Cubosome-delivered SIM affects the growth of HeLa spheroids. Three-dimensional HeLa cultures show significantly reduced viability when coincubated for 12 days with DOX-loaded MO-based cubosomes (+MO/DOX) and DOX:SIM-loaded MO-based cubosomes (+MO/DOX:SIM) compared with empty MO-based cubosomes (+MO). Phase-contrast representative images of the analyzed spheroids (left panel) and graphical plots summarizing the average area of the spheroids treated with designated formulations (right panel). Nontreated spheroids were used as a control. Magnification: 10× lens. Data are presented as mean ± SD; *p < 0.05. One-way ANOVA followed by the Bonferroni post hoc comparative test were performed.

Finally, to confirm the effect of the tested nanoparticles on the *in vitro* cell viability, we established three-dimensional HeLa cultures (spheroids) and exposed them to the tested phases (Figure 8). As expected, empty cubosomes did not affect the area and condition of the formed colonies, while both DOX-loaded and DOX:SIM-loaded cubosomes significantly reduced the area of the formed spheroids. Consequently, the observed anticancer effect was most effective when cubosome-delivered DOX was accompanied by SIM (Figure 8). These data serve as an additional confirmation of the cytotoxic properties of the DOX:SIM-loaded phases.

Collectively, the biological data indicate that cubosomes coloaded with the chemo drug and SIM are capable of effective suppression of the growth of cancerous cells.

## 4 Conclusions

The efficacy of combining simvastatin (SIM) with cytotoxic doxorubicin (DOX) for anticancer therapy has been previously described,^22−24^ but the reason for this enhancement and the operative mechanism during the simultaneous intracellular delivery of statins and doxorubicin were previously unknown.

In the Langmuir part of the study, we discuss very simple single component lipid monolayers, which possess selected properties of real biological membranes (POPC, fluidity; DMPS, negative charge), and the obtained results allow us to understand which factors determine the facility of DOX penetration into the lipid layer.

**Figure 9:**
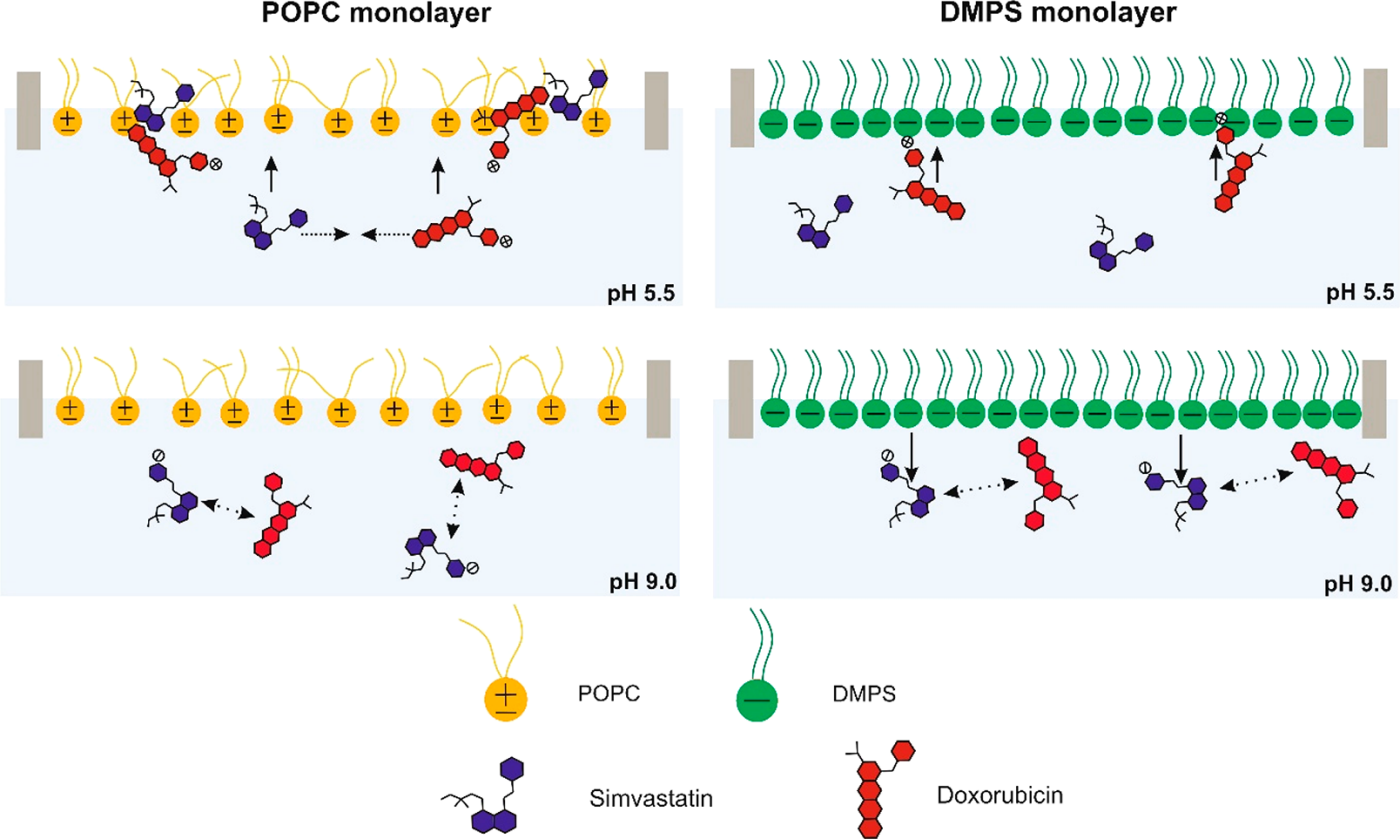
Schematic presentation of the DOX:SIM interactions with POPC and DMPS model membranes.

We demonstrate for the first time that the incorporation of positively charged DOX into neutral membranes is increased in the presence of SIM. The interaction of DOX with SIM and high affinity of the lipophilic SIM toward the lipid layers facilitate the introduction of both drugs into the zwitterionic (POPC) and uncharged (Chol) model monolayers (as schematically shown in Figure 9). Moreover, the penetration of both drugs is easier in the case of more liquid, neutral lipid layers such as POPC.

On the other hand, the negatively charged lipid, DMPS, interacts electrostatically with the positively charged DOX, which favors its introduction into the DMPS layer alone, and therefore, no improvement due to the simultaneous delivery of both drugs DOX and SIM from acidified and neutral solutions was observed for this lipid membrane.

The influence of the DOX interaction with SIM on the penetration of lipid layers explains the results of tests on cell lines. The effect of treatment of the designated model cell lines with single drugs and their combination confirmed that the coadministration of DOX and SIM can effectively reduce the viability of cancer cells. The analysis was further expanded by delivering SIM and DOX encapsulated in lipidic drug carriers, in this case cubosomes. The biological data indicate that cubosomes coloaded with the chemotherapeutic and the lipophilic statin, SIM, most effectively suppressed the growth of the cancerous cells.

## Supporting Information Available

Structural formula of drugs and molecules used to prepare the lipid monolayers and cubosomes (Figure S1); mean values of Langmuir isotherm parameters for lipid monolayers exposed to solutions of test molecules (Tables S1, S3, and S5); mean values of the increase in the molecular area read at π = 10 mN/m for the lipid monolayers formed on buffer containing test molecules (Tables S2, S4, and S6); representative graphs showing changes in surface pressure over time for lipid monolayers initially compressed to a surface pressure of 20 mN/m on buffer after injecting the test molecules (Figures S2–S4); mean values of the increase in the molecular area for lipid monolayers initially compressed to a surface pressure of 20 mN/m and left for 4 h on buffer containing test molecules (Tables S7–S9); representative graphs of the viability of HeLa, A172, and T98G cells treated for 24 and 48 h with empty cubosomes, doxorubicin-loaded cubosomes, and doxorubicin- and simvastatin-loaded cubosomes examined by the MTS-based assay (Figure S5) (PDF)
