# Supplementary material for: Simvastatin Coadministration Modulates the Electrostatically Driven Incorporation of Doxorubicin into Model Lipid and Cell Membranes
Source: ACS Biomater Sci Eng. 2022 Sep 29;8(10):4354–64. doi: 10.1021/acsbiomaterials.2c00724 (PMC9554873; doi:10.1021/acsbiomaterials.2c00724)
Supplement: Supplementary file 1 — ab2c00724_si_001.pdf [file ab2c00724_si_001.pdf]

Supporting Information

Simvastatin Co-administration Modulates Electrostatically Driven Incorporation of Doxorubicin into Model Lipid and Cell Membranes

Aleksandra Bartkowiak[1], Ewa Nazaruk[1], Ewa Gajda[2], Marlena Godlewska[2], Damian Gaweł[3], Elżbieta Jabłonowska[1], Renata Bilewicz[1*]

[1] Faculty of Chemistry, University of Warsaw, Pasteura 1, 02093 Warsaw, Poland

[2] Department of Biochemistry and Molecular Biology, Centre of Postgraduate Medical Education, Marymoncka 99/103, 01-813 Warsaw, Poland

[3] Department of Cell Biology and Immunology, Centre of Postgraduate Medical Education, Marymoncka 99/103, 01-813 Warsaw, Poland

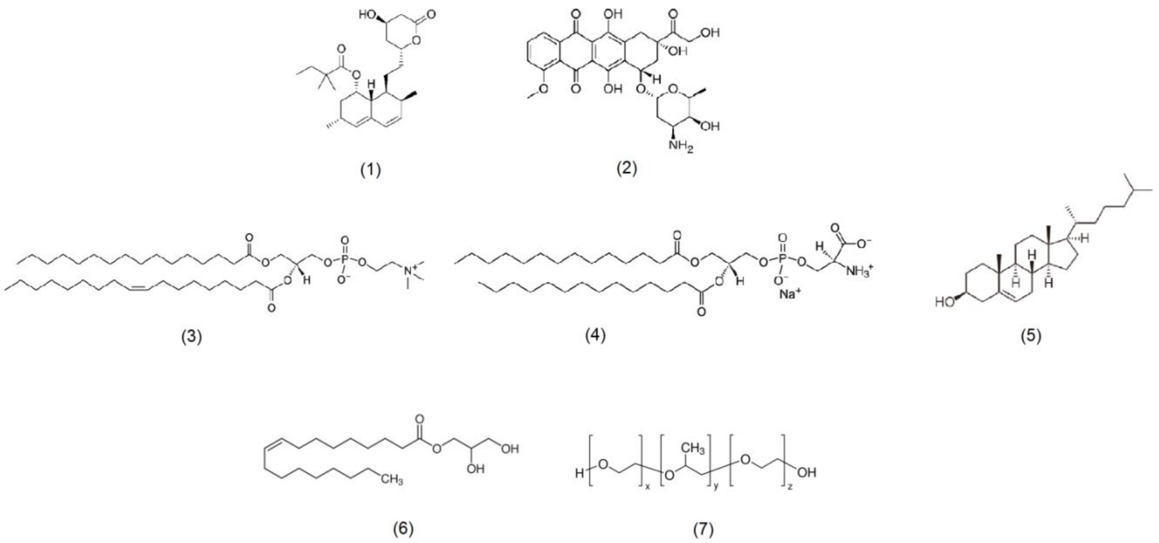

Figure S1. Structural formula of drugs: simvastatin (SIM, lactone) (1), doxorubicin (DOX) (2) and molecules used to prepare the lipid monolayers: POPC (3), DMPS (4), cholesterol (Chol) (5) and cubosomes: monoolein (MO) (6), Pluronic F108 (x=136, y=52) (7).

Table S1. The parameters of the $\pi - A$ isotherms for Langmuir monolayer of POPC exposed to solutions of DOX, SIM and their mixture DOX:SIM

Subphase	$A_{lift-off}$ [Å^2]	A_{coll} [Å^2]	π_{coll} [mN/m]	$A_{10\ mN/m}$ [Å^2]	$A_{20\ mN/m}$ [Å^2]	$maxC_s^{-1}$ [mN/m]
			pH 5.5			
**MES pH 5.5**	104.5±0.5	47.7±2.3	41.5±2.2	79.4±0.5	66.0±0.9	77±3.5
**DOX (10^{-6} M)**	114.1±3.9	48.0±1.0	40.7±0.3	82.5±1.1	67.7±1.0	69±1.2
**SIM (10^{-6} M)**	271.0±1.4	50.2±0.2	37.0±0.7	143.4±0.6	107.8±1.5	37±0.6
**DOX:SIM (1:1, 10^{-6} M)**	327.7±2.5	49.2±3.0	37.0±0.5	156.2±3.6	114.3±1.5	35±1.5
			pH 9.0			

**TRIS pH 9.0**	105.5±0.5	45.7±0.7	40.9±1.6	76.8±0.3	63.2±0.5	74±2.9
**DOX (10^{-6} M)**	117.2±1.3	50.4±0.2	40.2±0.7	85.3±2.0	69.7±1.1	70±2.5
**SIM (10^{-6} M)**	134.3±2.1	50.8±0.8	39.2±1.1	88.2±2.1	73.0±1.6	62±3.5
**DOX:SIM (1:1, 10^{-6} M)**	150.7±1.1	50.9±0.8	38.9±1.1	96.5±0.9	78.1±1.8	54±1.0

(mean ± SD, n=3)

Table S2. Increase in the molecular area read at π = 10 mN/m for the POPC monolayer formed on MES buffer (pH 5.5) and TRIS buffer (pH 9.0) containing DOX (10^{-6} M), SIM (10^{-6} M) and DOX:SIM molecules in the molar ratio 1:1.

Subphase	MES pH 5.5	TRIS pH 9.0
$\boldsymbol{\pi = 10\ mN/m}$		
ΔA_{DOX} (1×10^{-6}) [Å²/molecule]	3.1 ± 1.1	8.5 ± 2.0
ΔA_{SIM} (1×10^{-6}) [Å²/molecule]	64.0 ± 0.6	11.4 ± 2.1
$\Delta A_{DOX:SIM}$ $(1{:}1)$ [Å²/molecule]	**76.8 ± 3.6**	**19.7 ± 0.9**

Table S3. The parameters of the $\pi - A$ isotherms for Langmuir monolayer of DMPS exposed to solutions of DOX, SIM and their mixture DOX:SIM.

**Subphase**	$A_{lift-off}$ [Å²]	A_{coll} [Å²]	π_{coll} [mN/m]	$A_{10\ mN/m}$ [Å²]	$A_{20\ mN/m}$ [Å²]	$maxC_s^{-1}$ [mN/m]
pH 5.5						
**MES pH 5.5**	110.8±0.8	28.3±0.8	57.7±2.3	72.5±1.1	44.1±1.2	186±5.5
**DOX (10^{-6} M)**	148.5±3.0	25.7±0.4	57.3±2.4	103.3±0.5	82.1±0.9	100±4.5
**SIM (10^{-6} M)**	248.2±3.8	26.1±1.0	57.5±1.0	130.3±2.6	92.5±2.1	83±4.0
**DOX:SIM (1:1, 10^{-6} M)**	277.0±2.6	25.2±0.5	54.2±0.7	163.9±0.6	117.6±1.7	50±2.5
pH 9.0						
**TRIS pH 9.0**	110.0±2.0	27.5±1.8	63.5±1.4	69.6±1.8	45.9±1.5	149±4.4
**DOX (10^{-6} M)**	125.8±2.7	23.1±0.9	58.8±2.0	81.2±2.0	59.2±1.3	88±5.2
**SIM (10^{-6} M)**	127.5±3.5	25.0±2.0	60.2±1.6	76.9±2.1	50.1±1.2	110±5.0
**DOX:SIM (1:1, 10^{-6} M)**	148.0±3.1	23.4±1.8	63.7±0.9	91.4±2.6	64.2±1.5	92±3.6

(mean ± SD, n=3)

Table S4. Increase in the molecular area read at π = 10 mN/m for the DMPS monolayer formed on MES buffer (pH 5.5) and TRIS buffer (pH 9.0) containing DOX (10^{-6} M), SIM (10^{-6} M) and DOX:SIM molar ratio 1:1, 10^{-6} M

Subphase	MES pH 5.5	TRIS pH 9.0
$\boldsymbol{\pi = 10\ mN/m}$		
ΔA_{DOX} (1×10^{-6}) [Å²/molecule]	30.8 ± 0.5	11.6 ± 2.0
ΔA_{SIM} (1×10^{-6})	57.8 ± 2.6	7.3 ± 2.1

[Å^2/molecule]		
$\Delta A_{DOX:SIM}$ (1:1) [Å^2/molecule]	**91.4 ± 0.6**	**21.8 ± 2.6**

Table S5. The parameters of the $\pi - A$ isotherms for Langmuir monolayer of Chol exposed to solutions of DOX, SIM and their mixture DOX:SIM.

Subphase	$A_{lift-off}$ [Å^2]	A_{coll} [Å^2]	π_{coll} [mN/m]	$A_{10\ mN/m}$ [Å^2]	$A_{20\ mN/m}$ [Å^2]	$maxC_s^{-1}$ [mN/m]
			pH 5.5			
**MES pH 5.5**	45.0±0.8	37.9±1.6	44.0±1.9	42.3±0.8	40.5±1.5	400±9.0
**DOX (10^{-6} M)**	50±2.0	39.2±2.0	39.6±2.3	45.1±1.5	42.3±1.5	195±6.1
**SIM (10^{-6} M)**	185±5.5	50.4±2.5	44.0±2.5	108.4±3.4	76.2±2.5	86±5.0
**DOX:SIM (1:1, 10^{-6} M)**	194±3.5	54.0±1.2	43.5±2.1	117.4±2.0	85.1±2.0	82±5.5
			pH 9.0			
**TRIS pH 9.0**	45.4±2.1	38.0±1.0	43.5±1.8	42.4±1.3	40.3±1.0	397±7.0
**DOX (10^{-6} M)**	49.8±1.5	38.5±1.2	43.0±1.0	44.9±1.4	46.6±1.7	225±5.2
**SIM (10^{-6} M)**	62.5±1.8	39.3±0.8	42.5±2.0	46.6±1.2	43.1±0.8	223±6.4
**DOX:SIM (1:1, 10^{-6} M)**	63.8±2.4	42.0±2.1	42.8±1.6	48.4±2.0	44.1±2.0	195±4.1

*(mean ± SD, n=3)

Table S6. Increase in the molecular area read at π = 10 mN/m for the Chol monolayer formed on MES buffer (pH 5.5) and TRIS buffer (pH 9.0) containing DOX (10^{-6} M), SIM (10^{-6} M) and DOX:SIM molar ratio 1:1, 10^{-6} M).

Subphase	MES pH 5.5	TRIS pH 9.0
	$\boldsymbol{\pi = 10\ mN/m}$	
ΔA_{DOX} (1×10^{-6}) [Å^2/molecule]	2.8 ± 1.5	2.5 ± 1.4
ΔA_{SIM} (1×10^{-6}) [Å^2/molecule]	66.1 ± 3.4	4.2 ± 1.2
$\Delta A_{DOX:SIM}$ (1:1) [Å^2/molecule]	**75.1 ± 2.0**	**6.0 ± 2.0**

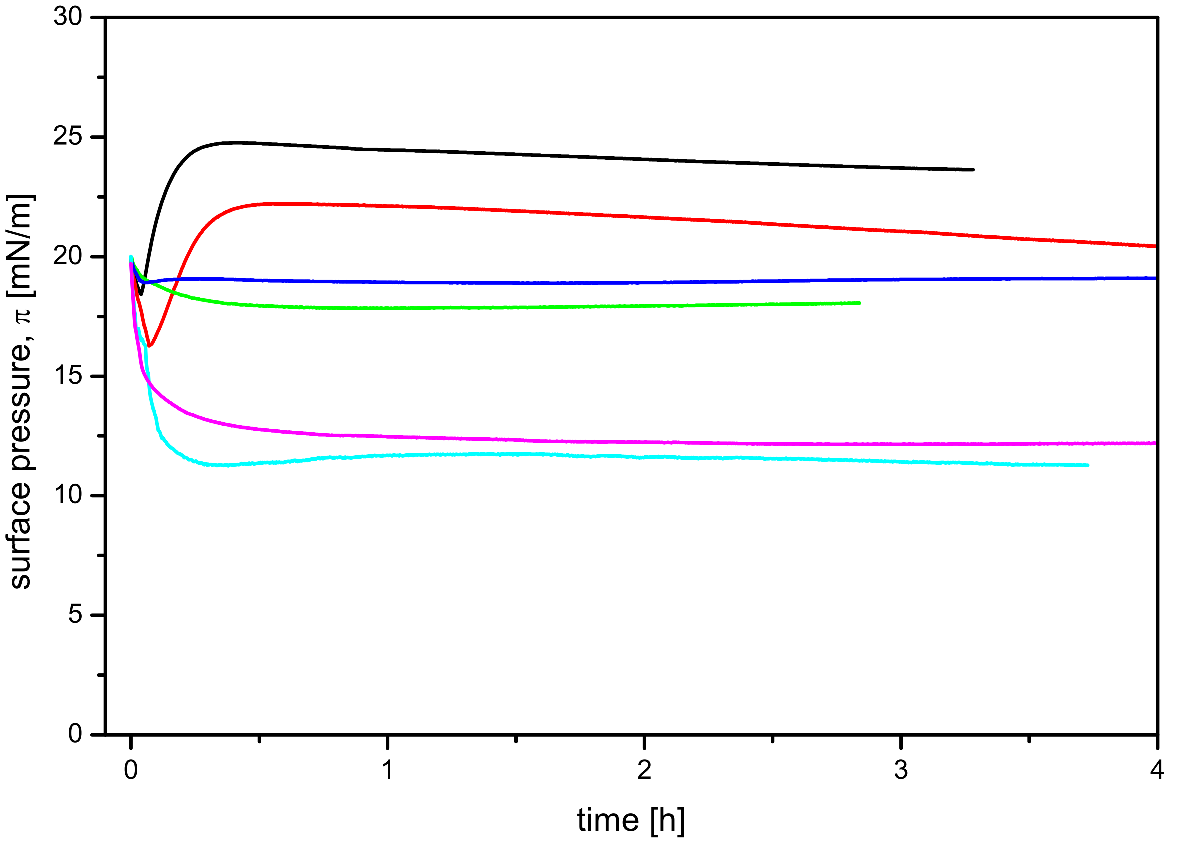

Figure S2. Changes in surface pressure over time for POPC, DMPS and Chol monolayers initially compressed to a surface pressure of 20 mN/m on MES and TRIS buffer after the injecting **DOX (10^{-6} M)**: DMPS, MES pH 5.5 (—), DMPS, TRIS pH 9.0 (—), POPC, MES pH 5.5 (—), POPC, TRIS pH 9.0 (—), Chol, MES pH 5.5 (—), Chol, TRIS 9.0 (—).

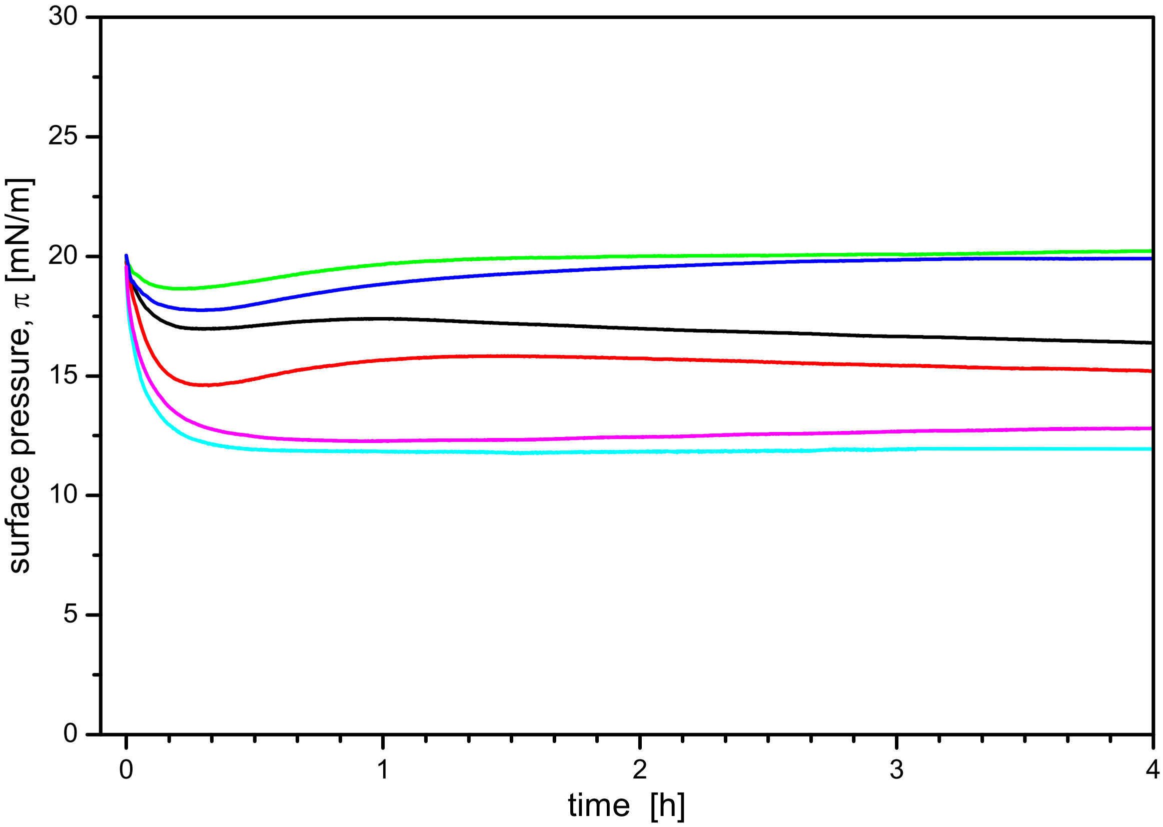

Figure S3. Changes in surface pressure over time for POPC, DMPS and Chol monolayers initially compressed to a surface pressure of 20 mN/m on MES and TRIS buffer after the **injecting SIM (10^{-6} M)**: DMPS, MES pH 5.5 (—), DMPS, TRIS pH 9.0 (—), POPC, MES pH 5.5 (—), POPC, TRIS pH 9.0 (—), Chol, MES pH 5.5 (—), Chol, TRIS 9.0 (—).

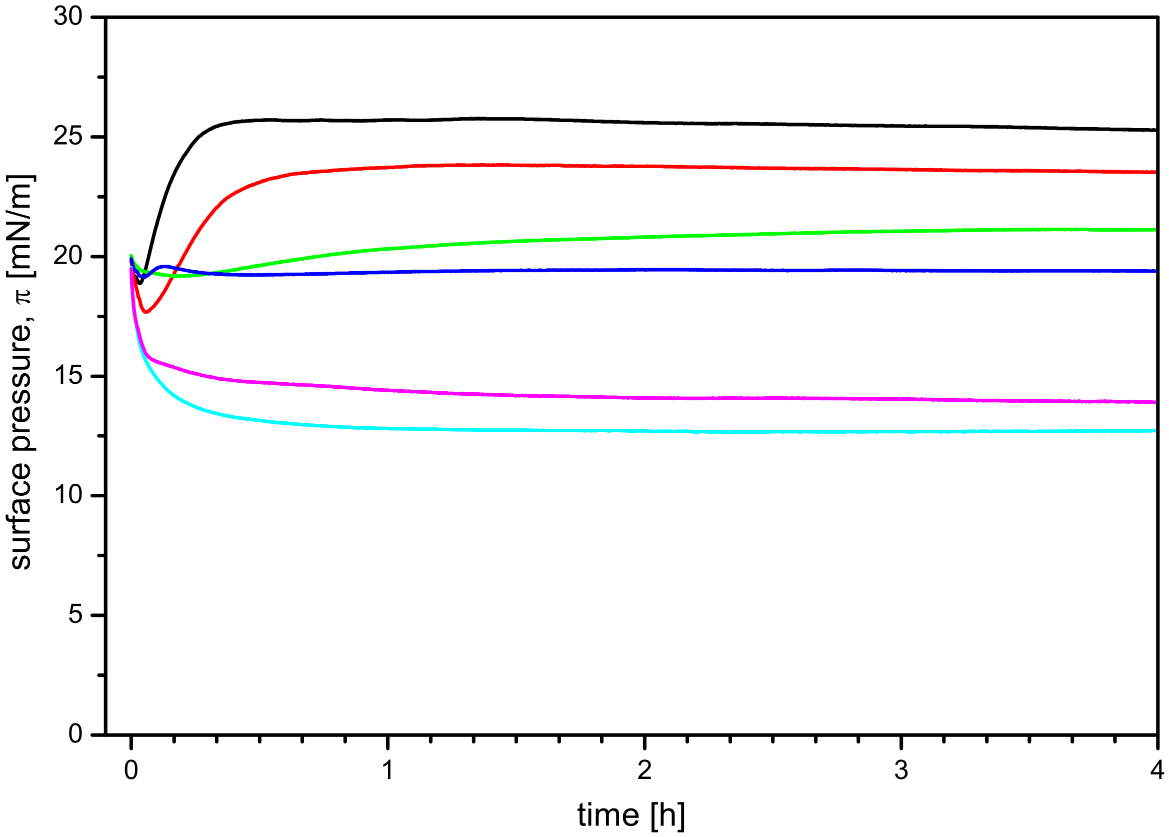

Figure S4. Changes in surface pressure over time for POPC, DMPS and Chol monolayers initially compressed to a surface pressure of 20 mN/m on MES and TRIS buffer after the injecting **DOX:SIM (1:1,** 10^{-6} M): DMPS, MES pH 5.5 (—), DMPS, TRIS pH 9.0 (—), POPC, MES pH 5.5 (—), POPC, TRIS pH 9.0 (—), Chol, MES pH 5.5 (—), Chol, TRIS 9.0 (—).

Table S7. POPC monolayers initially compressed to a surface pressure of 20 mN/m and left for 4h on MES and TRIS buffer containing DOX (10^{-6} M), SIM (10^{-6} M) and DOX:SIM (1:1 molar ratio, 10^{-6} M).

	MES pH 5.5	MES pH 5.5	TRIS pH 9.0	TRIS pH 9.0
	π	***Δπ***	***π***	***Δπ***
Buffer	14.2	0.0	14.0	0.0
DOX	18.0	3.8	19.0	5.0
SIM	20.1	5.9	19.8	5.8
DOX:SIM (1:1)	21.1	6.9	19.4	5.4

Table S8. DMPS monolayers initially compressed to a surface pressure of 20 mN/m and left for 4h on MES and TRIS buffer containing DOX (10^{-6} M), SIM (10^{-6} M) and DOX:SIM (1:1 molar ratio, 10^{-6} M).

	MES pH 5.5	MES pH 5.5	TRIS pH 9.0	TRIS pH 9.0
	π	$\Delta\pi$	π	$\Delta\pi$
Buffer	14.9	0.0	14.0	0.0
DOX	23.7	8.8	21.1	7.1
SIM	16.6	1.7	15.5	1.5
DOX:SIM (1:1)	25.5	10.6	23.6	9.6

Table S9. Chol monolayers initially compressed to a surface pressure of 20 mN/m and left for 4h on MES and TRIS buffer containing DOX (10^{-6} M), SIM (10^{-6} M) and DOX:SIM (1:1 ratio, 10^{-6} M).

	MES pH 5.5	MES pH 5.5	TRIS pH 9.0	TRIS pH 9.0
	π	$\Delta\pi$	π	$\Delta\pi$
Buffer	11.2	0	13.1	0.0
DOX	11.4	0.2	12.1	-1.0
SIM	11.9	0.7	12.7	-0.4
DOX:SIM (1:1)	12.7	1.5	14.0	0.9

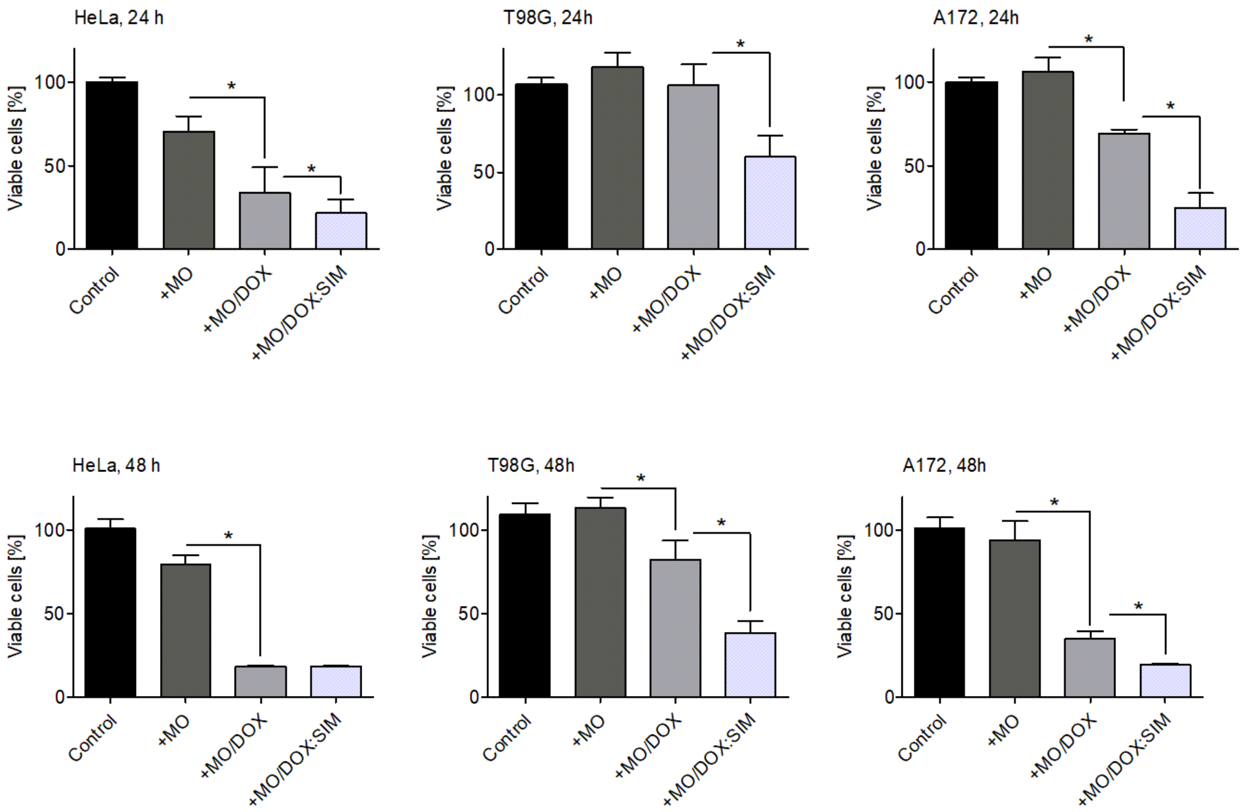

Figure S5. The viability of HeLa, A172 and T98G cells treated for 24 h (upper panel) and 48 h (lower panel) with empty cubosomes (+MO), doxorubicin-loaded cubosomes (+MO/DOX) and doxorubicin- and simvastatin-loaded cubosomes (+MO/DOX:SIM) determined by MTS-based assay. Treatment with MO/DOX:SIM results in a strong reduction in the cells' viability. Non-treated cells served as a control. Data are presented as mean ± standard deviation (SD). * $p < 0.05$.
